# Pruritus in Diabetes and Icterus

**Published:** 1885-05-20

**Authors:** Ernest Besnier

**Affiliations:** Hospital St. Louis, Paris


### PRURITUS IN DIABETES AND ICTERUS.

Clinical Lecture of DR. ERNEST BESNIER, Hospital St. Louis, Paris.

**Translated from the "Journal de Medicine" by Dr. E. Van Goidtsnoven, of Atlanta, Georgia.**

When there presents, in a middle-aged subject, violent and persistent itching, unaccompanied by any lesion, one may invariably feel authorized to entertain an unfavorable prognosis.

This condition was that of a man, 57 years old, under hospital treatment for a pruritus of the most intense character; and yet, notwithstanding the wildest and most painful scratchings, exhibited neither pigmentation nor the slightest tegumental alteration. This is due to the fact that in cases of this character the skin often opposes a great resistance, and there elapses a considerable time before pigmentation follows. At all events, such violent pruriti, without any apparent cause, should always evoke the idea of a dyscrasial affection, either of glycosuria or of albuminuria, etc. Such was precisely the case in this instance, although the exact time of the incipiency of the disease could not be determined. Sugar and albumine presented simultaneously in the urine; and it is probable that glycosuria antedated and ultimately called forth albuminuria. Hence, one should, as this fact indicates, think of examining the urine whenever pruritus presents without any lesion, especially if the patient be, as in this case, somewhat advanced in years.

This instance taught another lesson: Although the character of the affection seemed to indicate that it should be traced back to no less than a year, there was neither erythema nor any mark of balanoposthitis, whilst in the majority of cases glycosuria is made manifest through various accidents affecting the genital organs, a diagnostic point of the highest importance. The absence of these eruptions, so frequent in cases of this character, shows the important role performed by the individual constitution in the mode of reaction under morbific influences.

Pruritus without lesion is also frequently found in connection with icterus. In this instance there is no primary alteration of the skin; but the itching is so intense, that scratching often produces excoriations. That the presence of the coloring matter of the bile on the surface of the skin is the cause of the pruritus does not seem to be certain after all. Pruritus may appear even before icterus is manifested, as has been demonstrated by Graves; and it may persist after icterus has disappeared. One may admit, as a possible theory, that there exists amongst the elements of the absorbed bile other substances capable of irritating the external integument. One may also suppose that there possibly exists in this instance a nervous influence not unlike that seen in urticaria, the latter oftentimes manifesting itself almost immediately after the ingestion of such articles of food as ordinarily produce it.

Pruritus associated with icterus is felt over the surface of limbs, over the shoulders, the lateral parts of the trunk; over bony projections; over the plantar and palmar surfaces, and in the latter the thickness of the integuments prevents any lesion being effected through scratching. It is especially intense during the night, as is invariably observed in all forms of pruritus, whatever the source or origin may be.

This pruritus is extremely rebellious to all forms of treatment. Locally, if but a small surface is involved, it can be soothed through applications of flannel soaked in a solution of atropia containing 5 to 10 centigrammes of this substance in 20 grammes of water; the compress being covered over with oiled silk. One may use, also, acid or acetous lotions over the whole surface of the body, ointments either simple or containing a feeble quantity of carbolic acid (3 grammes of the acid in 100 grammes of glycerole of starch). Acetous baths (1 quart of vinegar to the bath) also greatly relieve the patient.

Internally, chloral and opium may oftentimes cause the pruritus to subside; but some patients illy bear these preparations, and not unfrequently get worse under their influence. These remedies should, therefore, be administered with caution. The seat of the disease should be the objective point, and the liver should, above all, engage particular attention. Milk diet and alkalies are especially indicated.

With respect to the general treatment of pruritus, we will simply call to mind that, since the anæsthetic influences of cocaine on the mucous membrane have been fully and universally acknowledged, Dr. Besnier has tried its application for local pruriti on surfaces where the skin assumes the mucous appearance, such as the anus, the vulva, the glans. Pruritus in these parts has been favorably modified through the agency of a pomade containing 1 or 2 grammes of cocaine in 40 grammes of excipient.

